# Significant liver fibrosis assessed using liver transient elastography is independently associated with low bone mineral density in patients with non-alcoholic fatty liver disease

**DOI:** 10.1371/journal.pone.0182202

**Published:** 2017-07-31

**Authors:** Gyuri Kim, Kwang Joon Kim, Yumie Rhee, Sung-Kil Lim

**Affiliations:** 1 Department of Internal Medicine, Yonsei University College of Medicine, Seoul, Republic of Korea; 2 Division of Endocrinology and Metabolism, Department of Medicine, Samsung Medical Center, Sungkyunkwan University School of Medicine, Seoul, Republic of Korea; Università degli Studi di Palermo, ITALY

## Abstract

**Background:**

Metabolic bone disorders frequently occur in patients with chronic liver disease; however, the association between liver fibrosis and bone mineral density in patients with non-alcoholic fatty liver disease (NAFLD) is unclear.

**Methods:**

This is a cross-sectional analysis of 231 asymptomatic subjects (160 women, 61.6 years old) from a university hospital setting, between February 2012 and December 2014. Bone mineral density (BMD) was measured at the lumbar spine, femur neck, and total hip using dual-energy X-ray absorptiometry (DXA). Liver fibrosis and steatosis were assessed using transient elastography.

**Results:**

Among a total of 231 individuals, 129 subjects (55.8%) had NAFLD. BMDs at lumbar spine, femur neck, and total hip were significantly lower in patients having NAFLD with significant fibrosis, compared with patients having NAFLD without significant fibrosis (*P*s<0.005). In patients with NAFLD, significant liver fibrosis revealed marked negative correlations with BMD at the lumber spine (r = –0.19, *P* = 0.032), femur neck (r = –0.19, *P* = 0.034), and total hip (r = –0.21, *P* = 0.016). A multivariate linear regression analysis revealed that significant liver fibrosis was independently correlated with low BMD at the femur neck (β = –0.18, *P* = 0.039) and total hip (β = –0.21, *P* = 0.005) after adjustment for age, sex, BMI, fasting plasma glucose, alanine aminotransferase, high-density lipoprotein cholesterol, and liver steatosis among patients with NAFLD. Using multivariable logistic regression, significant liver fibrosis was independently associated with overall osteopenia and osteoporosis in subjects having NAFLD (OR = 4.10, 95% CI = 1.02–16.45).

**Conclusion:**

The presence of significant liver fibrosis assessed via TE was independently associated with low BMD in NAFLD subjects.

## Introduction

Non-alcoholic fatty liver disease (NAFLD), one of the most common chronic metabolic liver disorders, is a major public health problem strongly correlated with growing prevalence of obesity or diabetes [1]. NAFLD ranges from simple steatosis to non-alcoholic steatohepatitis (NASH), representing progressive inflammation, which can progress to liver cirrhosis or hepatocellular carcinoma. Oxidative stress, pro-inflammatory cytokines, and lipotoxicity, in conjunction with various inflammatory reactions and fibrosis, have been found to be related with an increased risk of NASH and fibrosis progression [2]. Recently, liver fibrosis diagnosed based on liver biopsy rather than NAFLD activity score (NAS), a histological scoring system including features of steatosis, lobular inflammation, and hepatocellular ballooning but not fibrosis, was suggested to be the main crucial prognostic factor for overall and liver-related mortality in subjects with NAFLD [3,4].

Metabolic bone disease in patients with liver disease, known as hepatic osteodystrophy, is characterized by poor bone mineralization and loss of bone mass and quality, and its prevalence has also rapidly increased [5]. Several studies reported a degree of association between NAFLD and low bone mineral density (BMD), showing a higher risk of osteoporosis in postmenopausal women with NAFLD [6]. The association between NAFLD and osteopenia remains controversial. Furthermore, few studies have investigated whether NAFLD with liver fibrosis and low BMD are distinctively related to each other and whether significant liver fibrosis is an independent determinant of low BMD.

Osteoclasts and osteoblasts execute bone remodeling by resorbing and forming bone respectively, and physiological bone remodeling is tightly regulated by osteocytes. Multiple mediators, including oncofetal fibronectin, insulin-like growth factor-1 (IGF-1), the receptor activator of the nuclear factor kappa B ligand/osteoprotegerin (RANKL/OPG) pathway, and several inflammatory cytokines (e.g. tumor necrosis factor [TNF]-α, interleukin-6 [IL-6], and IL-17), are commonly involved in the process of bone loss by affecting the above cells and inducing an imbalance between bone formation and bone resorption in patients with liver disease [7–9]. In line with this, we hypothesized that progressive liver fibrosis in NAFLD indicating severe inflammatory status is associated with low BMD.

Chronic liver disease including NAFLD or liver cirrhosis assessed via abdominal ultrasonography or liver biopsy was reported to be associated with low bone mass [10–12]. However, abdominal ultrasonography often lacks the sensitivity required to identify early steatosis, and liver biopsy is an invasive procedure; performing liver biopsies on all at-risk individuals is not feasible in clinical practice [13]. To overcome this limitation, liver transient elastography (TE, Fibroscan) has been widely used as a non-invasive, easy, and rapid method for quantification and evaluation of liver fibrosis or steatosis [14,15]. Recently, several studies reported that liver stiffness measurement by using TE was a clinically useful noninvasive alternative to liver biopsy, providing a numerical value and well reflecting liver fibrosis [16,17]. In addition, simultaneous evaluation of hepatic steatosis by using the controlled attenuation parameter (CAP) based on TE has proven to be efficient in differentiating steatosis grades [18]. Therefore, in this present study, we identified patients with NAFLD and measured liver stiffness by using non-invasive TE in order to investigate the association between liver fibrosis and BMD in individuals with NAFLD.

## Materials and methods

### The study population

**Fig 1:**
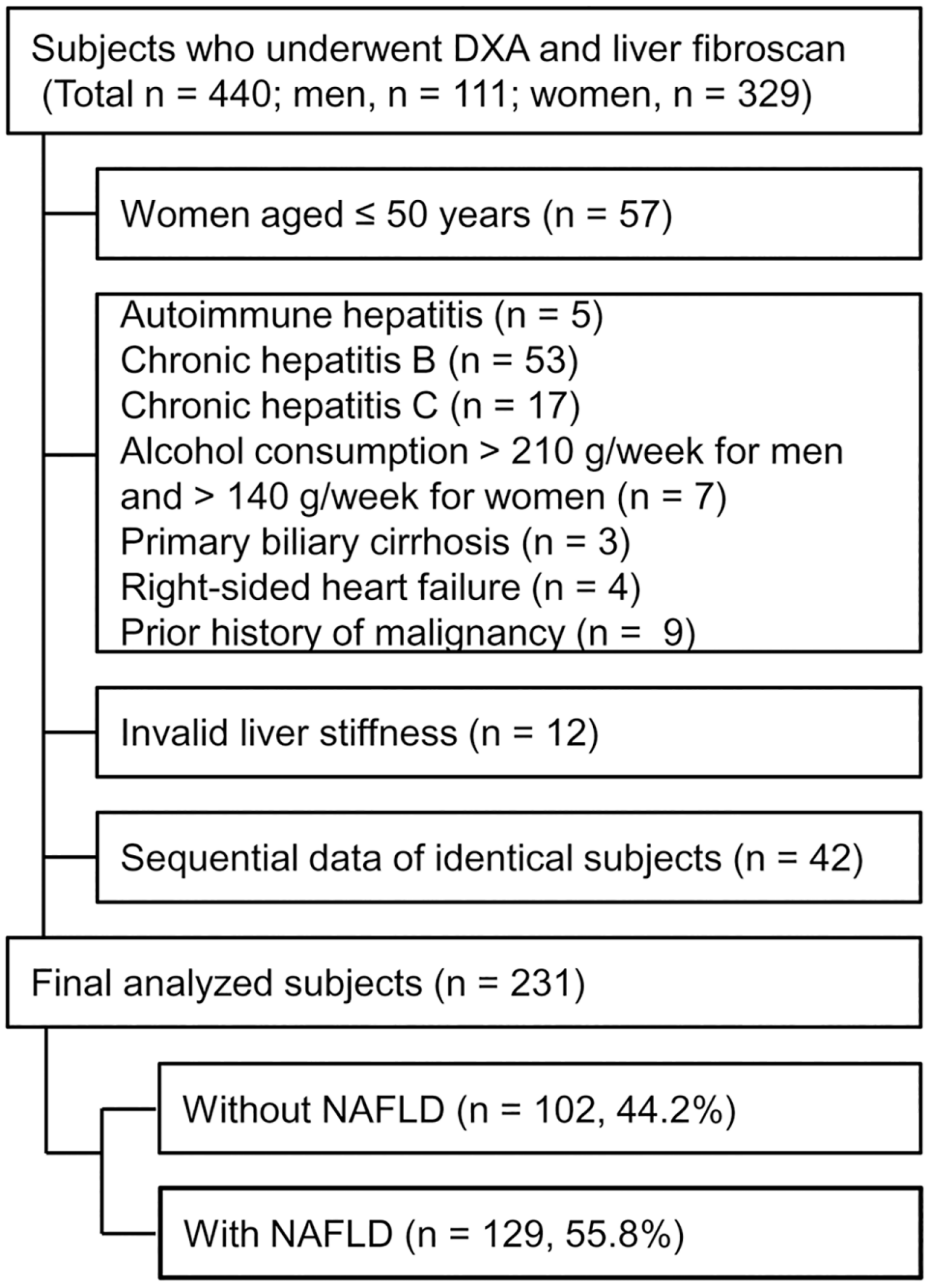
Study subject disposition.

This study retrospectively included 440 asymptomatic individuals who underwent evaluation via both dual-energy X-ray absorptiometry (DXA) and TE for a health check-up at the university-affiliated Severance Hospital, Seoul, Republic of Korea, between February 2012 and December 2014. As shown in Fig 1, exclusion criteria for this study were as follows: women aged ≤50 years (n = 57) to exclude pre-menopausal women, any etiological markers for chronic liver disease including positive serologic markers for hepatitis B virus (n = 53) and hepatitis C virus (n = 17), autoimmune hepatitis (n = 5), history of alcohol consumption >210 g/week for men and 140 g/week for women (n = 7), presence of primary biliary cirrhosis (n = 3), presence of right-sided heart failure (n = 4), prior history of malignancy (n = 9), invalid liver stiffness value (n = 12), and sequential data from identical subjects (n = 42). Ultimately, a total of 231 individuals were analyzed in this study. Informed consent for this study was not required because the database was only accessed for purpose of analysis without personal information. The protocol of this study was approved by the Institutional Review Board (IRB No. 4-2015-0285) of Severance Hospital.

### Clinical and laboratory variables

Body mass index (BMI) was defined as weight divided by the square of the height (kg/m^2^). Blood samples were obtained after an 8-hour fast. Routine serum chemistry determinations including plasma glucose, aspartate aminotransferase (AST), alanine aminotransferase (ALT), calcium, and albumin were performed using standard automated laboratory techniques; 25-hydroxyvitamin D was assayed using D3-radioimmunoassay-coated tubes (Biosource, Nivelles, Belgium). Glycated hemoglobin (HbA_1c_) was measured as previously described [19]. Plasma total cholesterol, triglycerides (TG), and high-density lipoprotein (HDL) cholesterol levels were measured using a Hitachi 7600 Auto Analyzer (Hitachi Instruments Service, Tokyo, Japan). Low-density lipoprotein (LDL) cholesterol was calculated using the Friedewald equation (LDL cholesterol [mg/dL] = total cholesterol [mg/dL]–HDL cholesterol [mg/dL] − TG [mg/dL] / 5) [20].

### Measurement of bone mineral density via DXA

Areal BMD was measured for the lumbar spine from L1 to L4, the femoral neck, and the total hip in all subjects using DXA (Hologic Delphi A version 12.6, Hologic, Waltham, MA, USA). Results are expressed as BMD (g/cm^2^) and T-scores. T-scores were calculated using reference values from the National Health and Nutrition Examination Survey (NHANES) III [21]. Osteoporosis was defined as a T-score of less than or equal to –2.5 at least one out of the three skeletal sites (lumbar spine, femur neck, or total hip) while osteopenia was between –1.0 and –2.5 at any one of these three sites, according to World Health Organization criteria [22].

### Measurement of liver stiffness and controlled attenuation parameter using TE

The measurement of liver stiffness (LS), CAP, and variability using TE was assessed as described previously [23–25]. Transient elastography (fibroscan) was performed by one experienced technician (more than 10,000 examinations), who was blind to patients’ clinical data [25]. The results are described as decibels per meter (dB/m) and kilopascals (kPa) for CAP and LS, respectively. The interquartile range (IQR) was assessed as an index of the intrinsic variability of LS values corresponding to the interval of LS values containing 50% of the valid measurements between the 25th and 75th percentiles. Only cases with at least 10 validated acquisitions, a success rate of at least 60%, and a ratio of the IQR of LS to median values (IQR/M_LS_) less than 0.3 were considered reliable [26]. The CAP was determined only when LS measurement was reliable for the simultaneous signals at the same volume of liver parenchyma, and the final CAP value was the median of individual CAP values using the same valid measurements [27]. In this study, a cut-off point of CAP ≥250 dB/m was defined as nonalcoholic fatty liver disease (NAFLD), and a cut-off point of LS >7.0 kPa was defined as significant liver fibrosis assessed via TE, based on previous studies [27,28].

### Statistical analyses

All continuous variables are expressed as mean ± standard deviation, and categorical variables are expressed as proportions. Differences were analyzed using independent Student’s *t*-tests or Mann-Whitney *U*-tests for continuous variables and chi-square tests or Fisher’s exact tests for categorical variables. Pearson’s correlation analysis was performed to examine the relationships between BMD and metabolic variables. A multivariate linear regression analysis was performed to determine the independent relationships of the studied variables, and standardized β was represented as the coefficient β. The odds ratios (ORs) and 95% confidence intervals (CIs) for significant liver fibrosis associated with overall osteopenia and osteoporosis were calculated using multivariate logistic regression analysis. In multivariate analysis, model 1 was a crude form and model 2 adjusted for age, sex, and BMI. In model 3, we adjusted for model 2 covariates in addition to fasting plasma glucose. Model 4 adjusted for age, sex, BMI, fasting plasma glucose, ALT, HDL cholesterol, and CAP. A *P* value of <0.05 was considered to be statistically significant. Statistical analyses were performed using PASW Statistics software, version 20.0 for Windows (SPSS Inc., Chicago, IL, USA).

## Results

### Baseline characteristics of the study population

The baseline clinical and laboratory characteristics of the total study population are shown in Table 1. For all subjects (n = 231), the mean age was 61.8 ± 8.4 years, and 160 (69.3%) were women. The mean BMI was 24.5 ± 3.3 kg/m^2^. A total 18.6% of patients had a history of diabetes (n = 43), and 15.4% had osteoporosis (n = 36). Of these 231 subjects, 129 (55.8%) had NAFLD. The subjects with NAFLD had significantly increased BMI, higher levels of HbA1c, fasting plasma glucose, ALT, albumin, TG, lower levels of HDL, and a higher proportion of significant liver fibrosis relative to subjects without NAFLD. In contrast, BMD at the lumbar spine, femur neck, or total hip showed similar values between subjects with and without NAFLD.

**Table 1:** Characteristics of study participants.

Variables	All (n = 231)	Subjects without NAFLD (n = 102, 44.2%)	Subjects with NAFLD (n = 129, 55.8%)	*P* value
Age (years)	61.8 ± 8.4	62.0 ± 8.8	61.6 ± 8.0	0.694
Sex (women), n (%)	160 (69.3)	70 (68.6)	90 (69.8)	0.482
BMI (kg/m^2^)	24.5 ± 3.3	22.9 ± 3.0	25.8 ± 3.1	<0.001
Diabetes mellitus, n (%)	43 (18.6)	17 (16.7)	26 (20.2)	0.308
Statin user, n (%)	72 (31.2)	24 (23.5)	48 (37.2)	0.018
Osteopenia, n (%)	109 (47.2)	48 (47.1)	61 (47.3)	0.973
Osteoporosis, n (%)	36 (15.6)	17 (16.7)	19 (14.7)	0.411
Overall osteopenia and osteoporosis	145 (62.8)	65 (63.7)	80 (62.0)	0.891
**Laboratory variables**				
HbA_1c_ (%)	6.3 ± 1.0	6.1 ± 0.7	6.5 ± 1.2	0.019
Fasting plasma glucose (mg/dL)	103.2 ± 21.4	100.0 ± 18.7	105.9 ± 23.2	0.036
AST (IU/L)	33.6 ± 31.4	34.6 ± 40.4	32.8 ± 21.9	0.660
ALT (IU/L)	31.8 ± 28.9	26.7 ± 26.6	35.7 ± 30.0	0.018
Calcium (mg/dL)	9.1 ± 0.5	9.1 ± 0.4	9.1 ± 0.5	0.606
Albumin (mg/dL)	4.2 ± 0.4	4.1 ± 0.5	4.2 ± 0.4	0.020
25-hydroxy vitamin D (IU)	22.7 ± 10.9	22.6 ± 10.4	23.0 ± 11.2	0.830
Total cholesterol (mg/dL)	174.8 ± 42.9	176.9 ± 49.3	173.1 ± 37.2	0.504
TG (mg/dL)	123.7 ± 69.8	105.8 ± 61.1	136.6 ± 73.0	0.001
HDL cholesterol (mg/dL)	49.2 ± 14.4	52.7 ± 17.0	46.5 ± 11.3	0.002
LDL cholesterol (mg/dL)	101.1 ± 32.3	103.9 ± 35.9	98.9 ± 29.1	0.278
**DXA scan**				
Lumbar spine BMD (g/cm^2^)	0.910 ± 0.174	0.896 ± 0.172	0.921 ± 0.176	0.280
Lumbar spine T-score	-0.87 ± 1.43	-0.97 ± 1.37	-0.79 ± 1.48	0.335
Femur neck BMD (g/cm^2^)	0.693 ± 0.124	0.685 ± 0.120	0.700 ± 0.128	0.365
Femur neck T-score	-1.11 ± 1.05	-1.19 ± 1.02	-1.05 ± 1.08	0.322
Total hip BMD (g/cm^2^)	0.838 ± 0.146	0.827 ± 0.139	0.847 ± 0.151	0.320
Total hip T-score	-0.35 ± 1.02	-0.45 ± 0.97	-0.27 ± 1.07	0.204
**Transient elastography**				
CAP (dB/m)	262.9 ± 51.2	217.0 ± 24.0	299.2 ± 35.3	<0.001
IQR_CAP_ (dB/m)	28.8 ± 13.0	30.7 ± 12.5	27.3 ± 13.3	0.056
Liver stiffness value (kPa)	5.70 ± 4.15	5.62 ± 4.94	5.76 ± 3.4	0.810
IQR_LS_ (kPa)	0.73 ± 0.62	0.72 ± 0.70	0.74 ± 0.55	0.754
IQR/median_LS_	0.13 ± 0.05	0.13 ± 0.05	0.13 ± 0.06	0.774
Significant liver fibrosis (>7 kPa), n, %	38 (16.5)	10 (9.8)	28 (21.7)	0.011

Values are presented as the mean ± standard deviation (SD) or n (%). AST, aspartate aminotransferase; ALT, alanine aminotransferase; BMD, bone mineral density; BMI, body mass index; CAP, controlled attenuation parameter; DXA, dual-energy X-ray absorptiometry; HDL, high density lipoprotein; IQR, interquartile range; LDL, low density lipoprotein; NAFLD, nonalcoholic fatty liver disease; TG, triglycerides.

**Fig 2:**
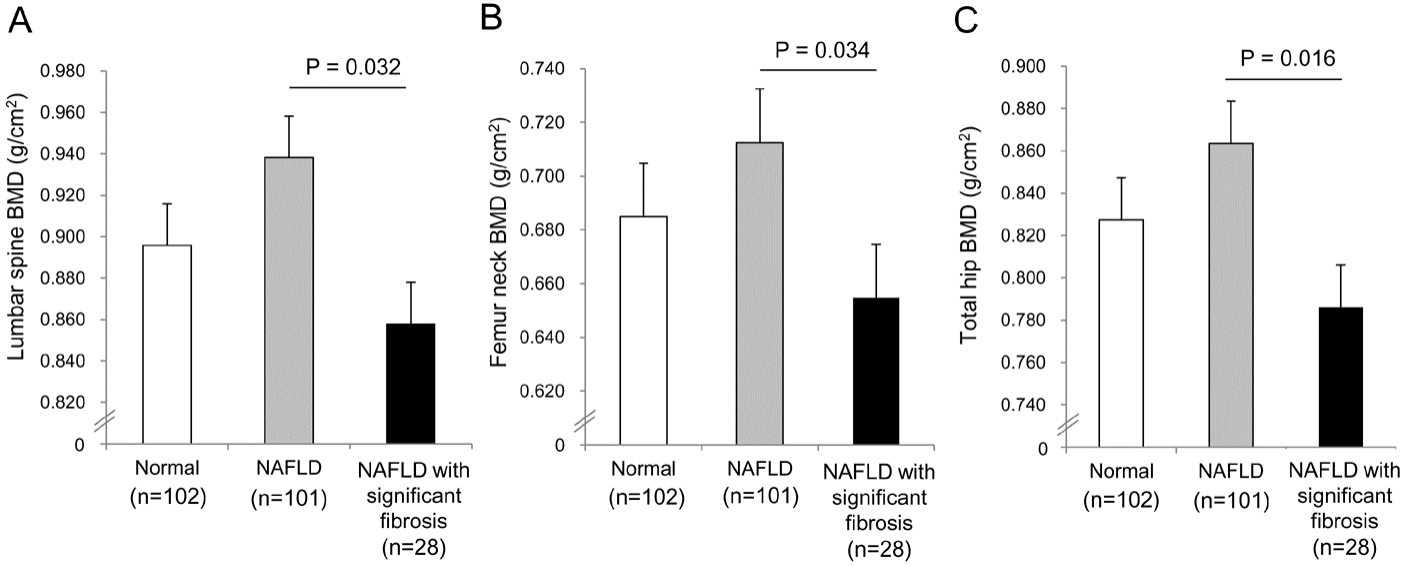
Bone mineral density at lumbar spine, femur neck, and total hip among subject with normal, NAFLD, and NAFLD with significant fibrosis. NAFLD, non-alcoholic fatty liver disease.

The characteristics of subjects with NAFLD according to significant liver fibrosis status are described in Table 2. Levels of serum AST, ALT, triglycerides, and CAP were significantly higher in subjects with significant liver fibrosis than in those without significant fibrosis, while lumbar spine, femur neck, and total hip BMDs were markedly lower. The prevalence of overall osteopenia and osteoporosis in NAFLD patients with significant fibrosis was significantly higher than in those without significant fibrosis (*P* = 0.010). Fig 2 displays BMDs at lumbar spine, femur neck, and total hip among subjects with normal, NAFLD, and NAFLD with significant fibrosis. BMDs were similar between normal and NAFLD groups but, patients with NAFLD with significant fibrosis showed significant lower BMDs, compared to normal or NAFLD group (*P*s<0.005).

**Table 2:** Comparison between subjects with and without significant liver fibrosis (LS >7 kPa) among subjects with NAFLD (n = 129).

	Without significant fibrosis (LS value ≤7 kPa, n = 101)	With significant fibrosis (LS value >7 kPa, n = 28)	*P* value
**Age (years)**	61.9 ± 7.4	60.6 ± 10.1	0.775
**Sex (women), n (%)**	67 (66.3)	23 (82.1)	0.081
**BMI (kg/m**^**2**^**)**	25.5 ± 3.0	26.6 ± 3.3	0.131
**Diabetes mellitus, n (%)**	32 (16.6)	7 (25.0)	0.161
**Statin, n (%)**	41 (40.6)	7 (25.0)	0.097
**Osteopenia, n (%)**	45 (44.6)	16 (57.1)	0.167
**Osteoporosis, n (%)**	12 (11.9)	7 (25.0)	0.127
**Overall osteopenia and osteoporosis, n (%)**	57 (56.4)	23 (82.1)	0.010
**HbA**_**1c**_ **(%)**	6.4 ± 1.2	6.6 ± 1.2	0.253
**Fasting plasma glucose (mg/dL)**	106.5 ± 24.3	103.6 ± 18.5	0.955
**AST (IU/L)**	29.0 ± 18.1	46.3 ± 28.7	<0.001
**ALT (IU/L)**	31.1 ± 27.1	52.5 ± 34.6	<0.001
**Calcium (mg/dL)**	9.0 ± 0.5	9.3 ± 0.5	0.011
**Albumin (mg/dL)**	4.2 ± 0.3	4.4 ± 0.6	0.006
**25-hydroxy vitamin D (IU)**	22.6 ± 11.2	24.2 ± 11.3	0.527
**Total cholesterol (mg/dL)**	174.0 ± 38.6	169.8 ± 32.1	0.491
**TG (mg/dL)**	129.3 ± 68.2	162.8 ± 84.2	0.022
**HDL cholesterol (mg/dL)**	47.3 ± 11.9	43.7 ± 8.5	0.256
**LDL cholesterol (mg/dL)**	98.9 ± 30.7	98.8 ± 22.7	0.997
**Lumbar spine BMD (g/cm**^**2**^**)**	0.938 ± 0.180	0.858 ± 0.145	0.045
**Lumbar spine T-score**	-0.64 ± 1.51	-1.32 ± 1.26	0.045
**Femur neck BMD (g/cm**^**2**^**)**	0.712 ± 0.130	0.655 ± 0.111	0.048
**Femur neck T-score**	-0.96 ± 1.09	-1.38 ± 1.00	0.100
**Total hip BMD (g/cm**^**2**^**)**	0.864 ± 0.151	0.786 ± 0.137	0.023
**Total hip T-score**	-0.16 ± 1.04	-0.69 ± 1.07	0.037
**CAP (dB/m)**	295.7 ± 34.0	312.0 ± 37.7	0.042
**Liver stiffness value (kPa)**	4.65 ± 1.13	9.74 ± 5.43	<0.001

Values are presented as the mean ± standard deviation (SD) or n (%). AST, aspartate aminotransferase; ALT, alanine aminotransferase; BMD, bone mineral density; BMI, body mass index; CAP, controlled attenuation parameter; HDL, high density lipoprotein; LDL, low density lipoprotein; LS, liver stiffness; NAFLD, nonalcoholic fatty liver disease; TG, triglycerides.

### Associations between BMD and metabolic measures

To assess the relationship between BMD and metabolic variables in patients with NAFLD, a univariate analysis was performed (Table 3). BMDs at all sites revealed strong inverse correlations with significant liver fibrosis (lumbar spine, r = –0.19, *P* = 0.032; femur neck, r = –0.19, *P* = 0.034; total hip BMD, r = –0.21, *P* = 0.016), while no significant association was found between liver steatosis and BMD.

**Table 3:** Factors associated with bone mineral density in subjects with NAFLD (n = 129).

Variables	Lumbar spine (g/cm^2^)		Femur neck (g/cm^2^)		Total hip (g/cm^2^)	
	r	*P* value	r	*P* value	r	*P* value
**Age (years)**	0.05	0.575	-0.20	0.022	-0.15	0.084
**Sex (male = 0, female = 1)**	-0.44	<0.001	-0.48	<0.001	-0.46	<0.001
**BMI (kg/m**^**2**^**)**	0.21	0.019	0.31	<0.001	0.31	<0.001
**Diabetes mellitus**	0.04	0.643	0.138	0.119	0.135	0.126
**HbA**_**1c**_ **(%)**	0.01	0.911	0.03	0.800	0.06	0.574
**Fasting blood glucose (mg/dL)**	0.22	0.013	0.21	0.021	0.22	0.014
**AST (IU/L)**	-0.24	0.006	-0.07	0.421	-0.09	0.306
**ALT (IU/L)**	-0.19	0.029	0.01	0.903	-0.01	0.977
**Calcium (mg/dL)**	-0.05	0.449	0.07	0.328	0.04	0.596
**Albumin (mg/dL)**	-0.16	0.068	0.03	0.760	-0.05	0.586
**25-hydroxy vitamin D (IU)**	-0.19	0.075	-0.17	0.110	-0.12	0.242
**Total cholesterol (mg/dL)**	-0.12	0.182	-0.01	0.968	-0.01	0.918
**TG (mg/dL)**	-0.04	0.684	0.11	0.217	0.09	0.342
**HDL cholesterol (mg/dL)**	-0.22	0.015	-0.19	0.046	-0.15	0.104
**LDL cholesterol (mg/dL)**	-0.09	0.334	-0.05	0.613	-0.07	0.437
**CAP (dB/m)**	-0.08	0.343	0.02	0.846	0.05	0.605
^a^**Liver stiffness value (kPa)**	-0.03	0.745	-0.06	0.469	-0.07	0.457
**Significant liver fibrosis (LS > 7kPa)**	-0.19	0.032	-0.19	0.034	-0.21	0.016

^a^log-transformed.

AST, aspartate aminotransferase; ALT, alanine aminotransferase; BMD, bone mineral density; BMI, body mass index; CAP, controlled attenuation parameter; HDL, high density lipoprotein; LDL, low density lipoprotein; LS, liver stiffness; NAFLD, nonalcoholic fatty liver disease; TG, triglycerides.

### Multivariate analysis

Among patients with NAFLD, multivariate linear regression analyses were performed to investigate the independent association between BMD and significant liver fibrosis (Table 4). After adjustment for age, sex, BMI, fasting plasma glucose, ALT, HDL cholesterol, and CAP (representing liver steatosis), significant liver fibrosis was independently associated with BMDs at the femur neck (β = –0.18, *P* = 0.039) and total hip (β = –0.21, *P* = 0.005) in patients with NAFLD.

**Table 4:** Multivariate linear regression analysis of the correlation between BMD and significant liver fibrosis in subjects with NAFLD (n = 129).

	Lumbar spine BMD (g/cm^2^)			Femur neck BMD (g/cm^2^)			Total hip BMD (g/cm^2^)		
	R^2^	β	*P* value	R^2^	β	*P* value	R^2^	β	*P* value
Model 1	0.028	-0.19	0.032	0.027	-0.19	0.034	0.037	-0.21	0.016
Model 2	0.214	-0.15	0.064	0.308	-0.17	0.025	0.288	-0.21	0.007
Model 3	0.230	-0.12	0.163	0.344	-0.18	0.020	0.332	-0.23	0.003
Model 4	0.189	-0.10	0.312	0.320	-0.20	0.028	0.369	-0.23	0.013

Model 1: crude; Model 2: adjusted for age, sex, BMI; Model 3: adjusted for age, sex, BMI, fasting plasma glucose; Model 4: adjusted for age, sex, BMI, fasting plasma glucose, ALT, HDL cholesterol, CAP. ALT, alanine aminotransferase; BMD, bone mineral density; BMI, body mass index; CAP, controlled attenuation parameter; HDL, high density lipoprotein; LS, liver stiffness; NAFLD, nonalcoholic fatty liver disease.

Multivariate logistic regression analyses were performed on significant liver fibrosis for overall osteopenia and osteoporosis in patients with NAFLD (Table 5). In the Model 1, the presence of significant liver fibrosis was associated with as increased risk for overall osteopenia and osteoporosis with an odds ratio of 3.55 (95% CI = 1.25–10.09). Subjects having NAFLD with significant fibrosis showed an independent association with overall osteopenia and osteoporosis (OR = 4.10, 95% CI = 1.02–16.45) in the model 5 adjusted for age, sex, BMI, fasting plasma glucose, ALT, HDL cholesterol, and CAP, compared to those having NAFLD without significant fibrosis.

**Table 5:** Multivariate logistic regression analysis of significant liver fibrosis for overall osteopenia and osteoporosis in subjects with NAFLD (n = 129).

Variables	OR	95% CI	*P* value
Model 1	3.55	1.25–10.09	0.017
Model 2	4.01	1.21–13.31	0.023
Model 3	3.67	1.06–12.70	0.040
Model 4	4.10	1.02–16.45	0.047

Model 1: crude; Model 2: adjusted for age, sex, BMI; Model 3: adjusted for age, sex, BMI, fasting plasma glucose; Model 4: adjusted for age, sex, BMI, fasting plasma glucose, ALT, HDL cholesterol, and CAP. ALT, alanine aminotransferase; BMD, bone mineral density; BMI, body mass index; CAP, controlled attenuation parameter; HDL, high density lipoprotein; LS, liver stiffness; NAFLD, nonalcoholic fatty liver disease; OR, odds ratio.

## Discussion

In the present study, we firstly applied liver TE (Fibroscan), a noninvasive tool for showing numerical values for liver steatosis based on CAP, and liver fibrosis based on LS, in order to investigate the association between liver fibrosis and osteopenia in patients with non-alcoholic fatty liver disease (NAFLD). We showed that BMD was reduced in subjects with significant liver fibrosis (LS >7 kPa) and that the presence of significant liver fibrosis was correlated with low BMDs at all sites, including the lumbar spine, femur neck, and total hip in patients with NAFLD. After adjustment for all confounding variables, significant liver fibrosis remained an independent determinant of low BMD at the femur among NAFLD patients. Furthermore, subjects having NAFLD with significant fibrosis were significantly associated with overall osteopenia and osteoporosis compared to those having NAFLD without significant fibrosis.

Liver fibrosis is the result of a massive accumulation of extracellular matrix and scar formation, eventually resulting in cirrhosis [29]. Osteoporosis is a common complication of liver cirrhosis, which is a progressive and severe chronic liver disease, and up to about 40% of patients with chronic liver disease may be at risk of bone fracture [7,30]. Previously, children with NASH had a significantly lower BMD than those with NAFLD who did not have NASH [31]. In addition, as suggestive of NASH, subjects with NAFLD showing elevated serum ALT and C-reactive protein (CRP) were associated with low BMD [32]. Xia et al. reported that there was a synergistic worsening of the BMDs in subjects with both NAFLD and elevated serum ALT [33]. However, the relationship of liver fibrosis and BMD in asymptomatic subjects with NAFLD is poorly understood. In this study, the significant fibrosis in NAFLD, indicating progression of inflammation in liver steatosis, was markedly associated with low BMD. The association between NAFLD and osteopenia also remains controversial. NAFLD was associated with low lumbar BMD and osteoporotic fractures in children and adults in several studies [6,11,34]. Meanwhile, in men yet not in women, the presence of NAFLD was associated with osteoporotic fractures in another study [11], and simple steatosis of the liver did not affect BMD [32]. In this present study, the presence or absence of NAFLD did not significantly affect BMD values. However, the multivariate regression analysis revealed that significant liver fibrosis, not CAP, which indicated the degree of liver steatosis, was an independent determinant of low BMD. Therefore, we suggest that NAFLD subjects with significant liver fibrosis should be concerned about the risk of low BMD.

Interestingly, the presence of significant liver fibrosis remained an independent risk factor for low BMD only at the femur after adjustment of all confounding factors. In a crude model (model 1), the presence of significant liver fibrosis was an independent factor for spinal BMD; however, it was not an independent factor after adjusting for confounding factors. We were unable to find an explanation for these discrepancies. However, in this context, previous studies showed that loss of BMD at the femur was greater than that at the lumbar spine in patients who underwent liver transplantation [35] as well as in those with alcoholic liver cirrhosis [36]. Compared to the controls, the thickness of the cortex was significantly thinner in patients with significant fibrosis, which might be explained by enhanced endocortical bone resorption [36]. Further study is required to explore the mechanism of preferential bone loss in cortical bone in patients with liver fibrosis.

The pathophysiology linking liver fibrosis to low bone mass has not been well established due to complex and multifactorial mechanisms. Pathogenic mediators, including fibronectin, IGF-1, the RANKL/OPG system, and several cytokines, such as TNF-α and the IL-6 family, have been suggested to play important roles in the pathogenesis of bone loss in chronic liver disease [30,37]. Lipotoxicity, reactive oxidative stress, activated hepatic macrophages releasing pro-inflammatory cytokines, and hepatocyte-derived extracellular vesicles released during lipotoxicity can modulate hepatic inflammatory/immune system leading to hepatic injury, NASH, and fibrosis [38,39]. In addition, hepatic inflammation and fibrosis are also linked to adipose inflammation and insulin resistance, through a release of inflammatory mediators such as TNF-α, IL-6, and monocyte chemoattractant protein-1 (MCP-1) from adipose tissue [40,41]. Therefore, our results showing a strong association between low BMD and significant liver fibrosis in NAFLD may be partly explained by the development of osteopenia through the systemic inflammation and insulin resistance observed in this disorder. Vitamin D deficiency may also contribute to worsening bone health in chronic liver disease [30,42]. Indeed, 92% of patients with chronic liver disease revealed vitamin D deficiency, although most had chronic hepatitis C [43]. Low vitamin D and high parathyroid hormone levels were independently associated with the presence of NAFLD [44]. In contrast, Goral et al. reported normal vitamin D levels in patients with liver cirrhosis [45], and no direct relationship between vitamin D levels and the severity of osteoporosis was detected [7,46]. Likewise, the current study demonstrated no significant difference in vitamin D levels according to the presence of significant liver fibrosis, and no significant correlation was found between BMD and the level of vitamin D. Taken together, more studies are required to elucidate the mechanism of liver fibrosis–associated bone loss.

The present study has several strengths. To our knowledge, this is the first study to investigate the association between BMD and liver fibrosis assessed via liver TE in patients with NAFLD. Therefore, we could measure liver fibrosis or steatosis using TE, and we were able to easily identify subjects who had liver steatosis and verify significant liver fibrosis without invasive investigation. Secondly, we excluded subjects with autoimmune hepatitis, chronic hepatitis B, chronic hepatitis C, alcoholic hepatitis, drug-induced hepatitis, primary biliary cirrhosis, right-sided heart failure, or a history of malignancy, which could have yielded LS values for fibrosis with a different distribution when assessed via TE [47,48].

The current study also has limitations. It was based on a cross-sectional study design, which made it difficult to determine a causal relationship between liver fibrosis and low BMD. Moreover, although liver TE has been used to assess fibrosis and steatosis, liver biopsies were not conducted in this study as a reference standard for diagnosis and assessment of the degree of fibrosis or steatosis. However, recent studies revealed that liver TE measurement is well correlated with the degree of fibrosis or steatosis assessed via biopsy [14,49]. There are also some issues that have to be taken into account when using TE. When interpreting results of TE, factors including obesity, presence of liver steatosis, and elevated levels of serum ALT may affect the results [50–52]. Also, as a substantial interoperator variability is presented, liver TE measurement made by the same operator with a high number of examinations for operator experience may reduce the variation [53,54]. In the present study, liver TE was performed by a single experienced operator that may enhance the reliability of CAP and liver stiffness. Additionally, we did not assess bone turnover markers. Previous studies suggested that decreased bone formation or increased bone resorption affect the pathogenesis of hepatic osteodystrophy [7,10,55]. Moreover, factors may have a link with bone mineralization including use of hormonal replacement therapy, Homeostasis Model Assessment of Insulin Resistance (HOMA-IR) index in relation to insulin resistance, or CRP as a marker of systemic inflammation were not accessed in the study due to lack of these variables [56–58].

## Conclusions

In conclusion, we confirmed that the presence of significant liver fibrosis assessed via liver TE (Fibroscan) was strongly associated with overall osteopenia and osteoporosis in asymptomatic individuals with NAFLD. Importantly, the presence of significant liver fibrosis was an independent determinant for low BMD in patients with NAFLD. Altogether, these finding indicated that the progression of hepatic inflammation and fibrosis might be closely related to bone loss. As advanced hepatic osteodystrophy is difficult to treat and could increase the burden on society with adverse long-term prognosis and increased morbidity, early screening and assessment for the risk of bone loss, even in asymptomatic NAFLD patients with significant liver fibrosis, must be considered. Furthermore, prospective studies are warranted to confirm the causative relationship between the presence of significant fibrosis and the development of bone loss.
